# Inhaled Corticosteroids and the Risk of Lung Cancer in Patients with Bronchiectasis

**DOI:** 10.3390/jcm14217654

**Published:** 2025-10-28

**Authors:** Chaiyoung Lee, Ga Young Lee, Jiyoung Shin, Ji Young Lee, Jin Hwa Lee

**Affiliations:** 1 Division of Pulmonary and Critical Care Medicine, Department of Internal Medicine, College of Medicine, Ewha Womans University, Seoul 07804, Republic of Korea; chaiyoung@ewha.ac.kr (C.L.);; 2 Department of Health Care Policy Research, Korea Institute for Health and Social Affairs, Sejong 30147, Republic of Korea; 3 Inflammation-Cancer Microenvironment Research Center, College of Medicine, Ewha Womans University, Seoul 07804, Republic of Korea

**Keywords:** bronchiectasis, corticosteroid, lung cancer, incidence

## Abstract

**Background/Objectives**: Prescribing inhaled corticosteroids (ICS) to patients with bronchiectasis may be controversial. Studies investigating the association between ICS and the risk of lung cancer in patients with bronchiectasis are rare. **Methods**: Patients with bronchiectasis were enrolled from the National Sample Cohort Data of the National Health Insurance Service (NHIS-NSC) in Korea. Among them, patients with lung cancer were selected as the case group, and the control group was selected by matching the patient group with the propensity score at 1:5 for age, sex, and recruitment year. Kaplan–Meier analysis and Cox proportional hazards model analysis were performed to determine the risk of lung cancer associated with ICS use in patients with bronchiectasis. In addition, ICS dose–response analysis was performed to determine the risk of lung cancer in patients with bronchiectasis. **Results**: A total of 19,043 patients with bronchiectasis were included in the study. In patients with bronchiectasis, ICS use was associated with an increased risk of lung cancer. After adjusting for age, sex, pack-years of smoking, body mass index (BMI), household income, region of residence, and Charlson comorbidity index (CCI), ICS use was found to be significantly associated with an increased risk of lung cancer (aHR 1.40, 95% CI 1.17 to 1.67). Furthermore, we found that the cumulative incidence of lung cancer increased with the cumulative dose of ICS in patients with bronchiectasis. Subgroup analysis of lung cancer risk in patients with bronchiectasis using ICS showed that the risk of lung cancer was significantly higher in those aged 70 years or older, male, with a BMI of 23 kg/m^2^ or higher, with a history of smoking, with a higher number of pack-years of smoking, and with a higher CCI. **Conclusions**: In patients with bronchiectasis, the use of ICS is associated with an increased risk of lung cancer, which is affected by the cumulative dose of ICS.

## 1. Introduction

Bronchiectasis is a chronic respiratory disease characterized by irreversible airway dilation and persistent inflammation, leading to recurrent infections and progressive airway damage [1]. This chronic inflammatory state may increase the risk of developing lung cancer. Indeed, nationwide cohort studies have shown that patients with bronchiectasis have a higher risk of developing lung cancer than the general population [2,3]. This association supports the hypothesis that chronic inflammation plays a critical role in carcinogenesis.

As chronic inflammation is increasingly recognized as a major contributor to lung cancer development, there is growing interest in the use of anti-inflammatory treatments as a preventive strategy. Inhaled corticosteroids (ICS), known for their potent anti-inflammatory properties, have been associated with a reduced risk of lung cancer in patients with chronic obstructive pulmonary disease (COPD) in several studies [4,5,6,7].

However, whether similar benefits can be expected in bronchiectasis remains uncertain, since the inflammation profile and pathophysiological mechanisms of bronchiectasis differ substantially from those of COPD. Accordingly, current clinical guidelines do not recommend the use of ICS in patients with bronchiectasis, mainly due to their limited efficacy against neutrophilic inflammation, the potential for increased infection risk, and the lack of robust evidence supporting long-term clinical benefit [8,9]. Moreover, the immunosuppressive effects of ICS may theoretically contribute to oncogenesis, particularly in chronically inflamed and structurally compromised airways [10]. Therefore, while it is plausible that ICS use could influence the risk of lung cancer in patients with bronchiectasis, this association remains insufficiently investigated.

Therefore, this study aims to elucidate the impact of ICS therapy on lung cancer incidence among patients with bronchiectasis by analyzing data from a large, nationally representative cohort.

## 2. Materials and Methods

### 2.1. Study Population

The National Health Insurance Service (NHIS) operates a comprehensive claims database encompassing universal healthcare coverage for the Korean population. The National Sample Cohort (NSC) of NHIS represents a population-based longitudinal dataset derived from systematic random sampling of beneficiaries, spanning from 2002 through 2019, and serves as a valuable resource for epidemiological research and health outcomes studies. This study included all subjects aged 35 years or older and excluded those with a prior diagnosis of lung cancer before bronchiectasis. To minimize the inclusion of prevalent cases, patients diagnosed with bronchiectasis during the washout period (2002–2003) were also excluded.

Study participants with bronchiectasis and lung cancer were ascertained through systematic case identification using diagnostic classification codes from the International Classification of Diseases, Tenth Revision (ICD-10), encompassing both principal and secondary diagnostic positions. Bronchiectasis was defined as patients treated with ICD-10 code J47.x, and lung cancer was defined as patients diagnosed with ICD-10 code C34.x. ICS use was defined as any prescription of ICS, either as monotherapy or as part of combination inhalers. Patients with at least two prescriptions within a year were classified as ICS users. Standardized dose equivalencies, with references doses established as ciclesonide 32 mg, fluticasone 50 mg, beclomethasone hydroflualkan 50 mg, budesonide 80 mg, beclomethasone 100 mg, triamcinolone 200 mg, and flunisolide 200 mg. The cumulative ICS dose was categorized into three groups based on the 33.3 percentile cutoff: low dose (<43,751 μg), intermediate dose (>43,751 μg and <218,750 μg), and high dose (>218,750 μg), to more accurately characterize dose–response relationships and identify distinct outcome patterns. The risks were then compared between each group.

Cases were characterized as individuals with newly identified bronchiectasis who later developed lung cancer during follow-up. A control cohort comprising bronchiectasis patients without lung cancer was assembled through 1:5 propensity score-matching methodology, incorporating matching variables of age, sex, and enrollment year to ensure balanced comparison groups. Nearest-neighbor matching without replacement was applied, with a caliper width set at 0.2 standard deviations of the logit of the propensity score to optimize group balance.

The study protocol received approval from the Institutional Review Board (IRB) of Ewha Womans University Medical Center, Seoul, South Korea (IRB number: SEUMC2022-12-046). The IRB waived the requirement for informed consent due to the retrospective design of the study. All study protocols adhered strictly to the principles and guidelines set forth in the latest revision of the Declaration of Helsinki.

### 2.2. Statistical Analysis

Baseline characteristics of the study population were summarized as mean  ±  standard deviation for continuous variables and as frequency (percentage) for categorical variables. Group comparisons were performed using Student’s *t*-test for continuous data and the chi-square test for categorical data. The cumulative incidence of lung cancer in bronchiectasis cohorts with and without ICS exposure was evaluated by Kaplan–Meier curves, and intergroup differences were tested using the log-rank method. To analyze the risk of lung cancer according to ICS prescription, Cox regression model was used after adjusting for available confounding variables such as age, sex, smoking status, household income, residential area, body mass index (BMI), and Charlson comorbidity index (CCI). The CCI is a validated measurement tool that assigns weighted scores to 19 comorbid conditions, including cardiovascular disease, diabetes, and rheumatic disease, based on their predictive value for patient survival, and calculates a total score accordingly. Comorbidities such as rheumatoid diseases and immune system-related disorders, which may necessitate long-term corticosteroid therapy and potentially confound the relationship, were accounted for through CCI adjustment. This analytic approach was adopted to minimize bias and improve the validity and reliability of the study findings. Hazard ratio (HR) with 95% confidence interval (CI) was calculated for the risk of lung cancer. In addition, subgroup analysis was performed for patients with bronchiectasis who received ICS by stratifying strong confounding variables of lung cancer. Large-scale data processing utilized SAS 9.4 (SAS Institute, Cary, NC, USA), and subsequent analyses were carried out in Stata 16 (StataCorp, College Station, TX, USA) and R version 4.0.3 (R Foundation). All hypothesis tests were two-tailed with a significance threshold of *p* < 0.05.

## 3. Results

**Figure 1:**
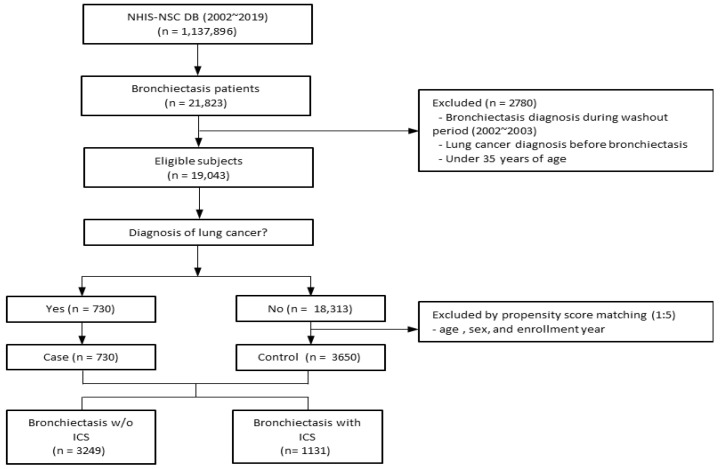
Overview of the participant inclusion process for the bronchiectasis cohort. Abbreviations: ICS, inhaled corticosteroids; NHIS-NSC, national sample cohort of National Health Insurance Service; w/o, without.

A total of 21,823 patients with bronchiectasis were extracted from the NHIS-NSC database. Exclusion criteria were age 35 years or younger, patients diagnosed with bronchiectasis during the wash-out period (2002–2003), and those with lung cancer before the diagnosis of bronchiectasis, a total of 2780 patients. Follow-up continued until either the development of lung cancer or the study end date on 31 December 2019, whichever happened first. Among 19,043 eligible subjects, there were 730 patients diagnosed with lung cancer, and a control group without lung cancer was selected using 1:5 propensity score matching. A total of 4380 patients were selected, including 3650 patients without lung cancer and 730 patients with lung cancer. Of these, 3249 patients with bronchiectasis were not prescribed ICS and 1131 were prescribed ICS (Figure 1).

Table 1 summarizes the demographic characteristics of the subjects who were not prescribed ICS and those who were prescribed ICS. The mean age of subjects prescribed ICS was 81 years, which was higher than that of those not prescribed ICS. The average BMI of the group prescribed ICS was 23.6 kg/m^2^, and the average pack-years of smoking was 10.7 pack-years, which were higher than non-ICS group. The ICS group was more likely to reside in rural areas and have a CCI of 1 or higher than the non-ICS group. The ICS group had more insurance claims with COPD or asthma disease codes at least twice during the study period than the non-ICS group.

**Figure 2:**
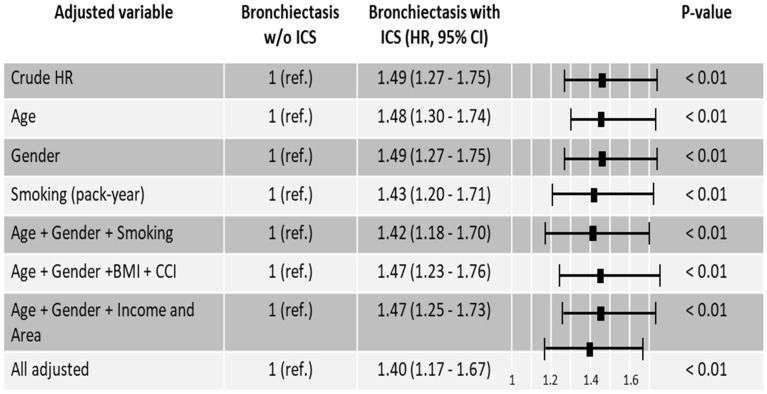
Hazard ratios for lung cancer in bronchiectasis patients with and without inhaled corticosteroids. Abbreviations: Area, residential area; BMI, body mass index; CCI, Charlson Comorbidity index; HR, Hazard ratio; ICS, inhaled corticosteroids; Income, household income; ref., reference; w/o, without.

In patients with bronchiectasis, ICS use was associated with an increased risk of lung cancer (HR 1.49, 95% CI 1.27 to 1.75). After covariate adjustment for age, sex, smoking pack-years, BMI, household income, residential region, and CCI, ICS exposure remained independently associated with elevated lung cancer risk (aHR 1.40, 95% CI 1.17–1.67) (Figure 2).

**Figure 3:**
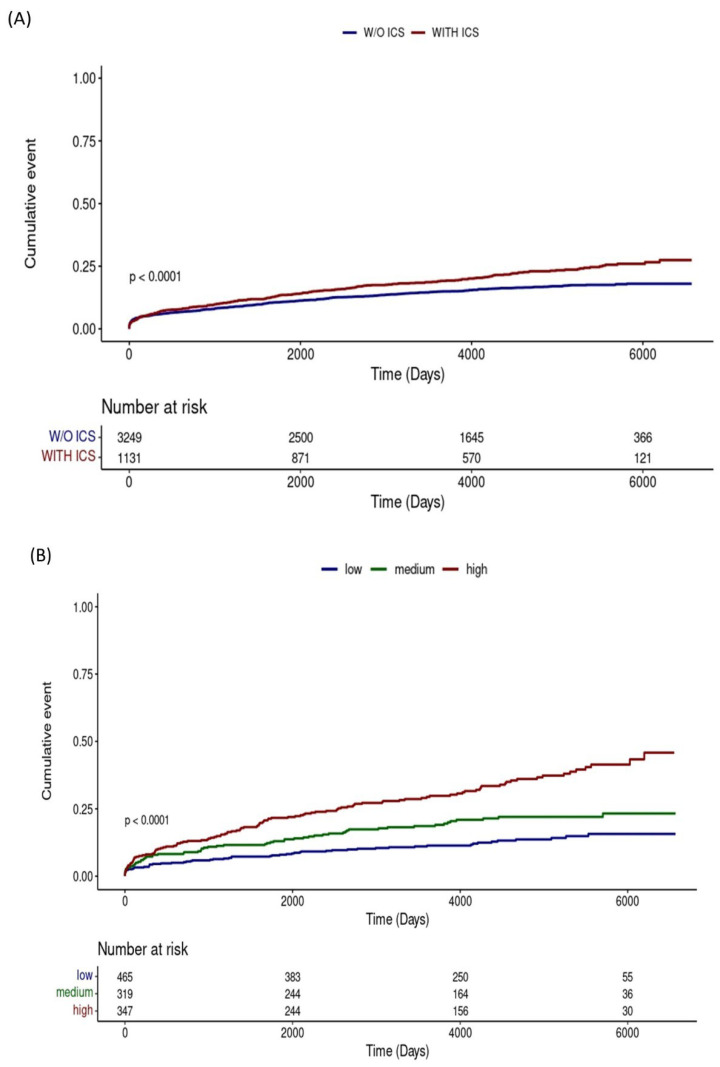
Kaplan–Meier curves for cumulative lung cancer incidence: (**A**) Comparison between patients with and without inhaled corticosteroids use; (**B**) Comparison across groups stratified by cumulative inhaled corticosteroids dose. Abbreviations: ICS, inhaled corticosteroids; w/o, without.

The Kaplan–Meier analysis in Figure 3A showed that the cumulative lung cancer incidence was higher in patients who used ICS than in those who did not use ICS (*p* < 0.001). The results of the analysis, divided into three groups according to the cumulative ICS dose, also showed that the higher the cumulative dose, the higher the incidence of lung cancer (Figure 3B, *p* < 0.001).

**Figure 4:**
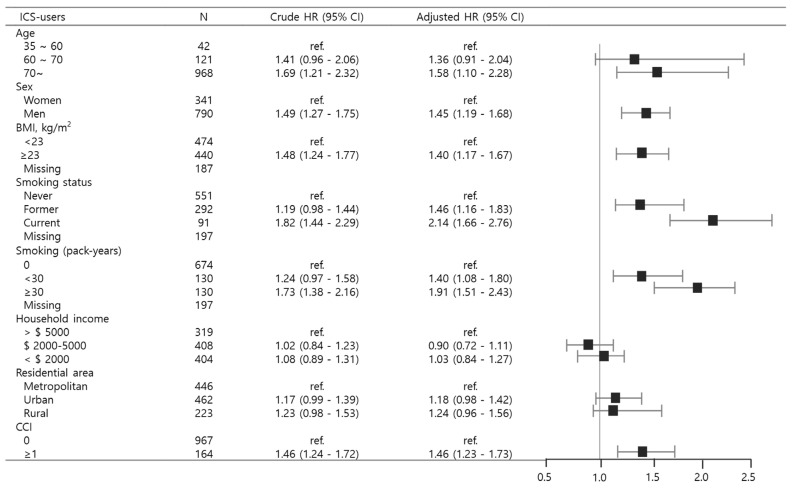
Subgroup analyses depicting lung cancer risk among bronchiectasis patients stratified by inhaled corticosteroid use. Abbreviations: BMI, body mass index; CCI, Charlson Comorbidity Index; HR, Hazard ratio; ICS, inhaled corticosteroids; ref., reference.

In patients with bronchiectasis using ICS, subgroup analysis was performed to determine whether there are differences in lung cancer risk by age, sex, BMI, smoking, household income, area of residence, and CCI. Individuals aged 70 or above exhibited a significantly higher risk of lung cancer (aHR 1.58, 95% CI 1.10–2.28), and the male population also demonstrated an increased hazard compared to females (aHR 1.45, 95% CI 1.19–1.68). The risk of lung cancer was significantly elevated in participants with BMI ≥ 23 kg/m^2^ (aHR 1.40; 95% CI 1.17–1.67), current smokers (aHR 2.14; 95% CI 1.66–2.76), those with smoking exposure ≥ 30 pack-years (aHR 1.91; 95% CI 1.51–2.43), and individuals with CCI ≥ 1 (aHR 1.46; 95% CI 1.23–1.73) as illustrated in Figure 4.

## 4. Discussion

This study used the NHIS-NSC data, the largest population-based cohort in Korea, and demonstrated that patients with bronchiectasis who used ICS had a significantly increased risk of lung cancer compared to non-ICS users, even after adjusting for potential confounders. Notably, higher cumulative doses of ICS were associated with a greater risk. To our knowledge, this is the first study to examine the dose-dependent association between ICS use and lung cancer risk in patients with bronchiectasis using nationwide real-world cohort data. Subgroup analysis further revealed that the increased risk was more pronounced in older individuals, males, and those with higher BMI or a history of smoking.

Several studies have demonstrated a protective association between ICS use and lung cancer risk in patients with COPD. In a large population-based cohort, Parimon et al. first reported that ICS exposure was associated with a significantly reduced risk of lung cancer among COPD patients [4]. This finding was subsequently supported by a Korean nationwide study by Lee et al., which showed a dose dependent reduction in lung cancer risk with increasing cumulative ICS exposure [5]. Similarly, Raymakers et al. confirmed this inverse association in a Canadian population-based cohort [6], and a recent meta-analysis by Tareke et al. further consolidated the evidence, demonstrating a consistent reduction in lung cancer risk associated with ICS use [7]. Collectively, these studies suggest that the anti-inflammatory and immunomodulatory effects of ICS may play a role in attenuating inflammation driven carcinogenesis in COPD. However, whether similar protective effects exist in patients with bronchiectasis remains unclear. Our finding indicated that ICS use in this population may be associated with an increased risk of lung cancer. These findings suggest that the effect of ICS on lung cancer risk may vary depending on the underlying airway disease. This divergence likely reflects key differences in inflammatory patterns and corticosteroid responsiveness between the COPD and bronchiectasis.

In COPD, lung carcinogenesis is primarily driven by smoking-related airway injury and chronic inflammation [11,12,13]. The COPD airway is characterized by elevated levels of pro-inflammatory cytokines such as IL-6 and IL-8, and by increased immune cell infiltration, contributing to a tumor-promoting microenvironment [14,15,16,17]. ICS mitigate these processes by downregulating inflammatory gene expression and suppressing immune cell recruitment. Their efficacy is particularly evident in steroid-responsive COPD phenotypes with eosinophilic inflammation. Through attenuation of airway inflammation and oxidative stress, ICS may help reduce DNA damage and epithelial remodeling, thereby limiting malignant transformation [18,19,20].

In contrast, bronchiectasis is associated with an inherently higher risk of lung cancer due to its distinct pathogenesis involving chronic bacterial infection, persistent neutrophilic inflammation, and airway remodeling with fibrosis [21,22,23]. Unlike the mixed inflammatory profile of COPD, bronchiectasis is dominated by neutrophilic inflammation, which is poorly responsive to corticosteroids [24,25]. Moreover, ICS may suppress key components of host immunity, including macrophage and T-cell function, thereby increasing vulnerability to infections such as non-tuberculous mycobacteria. Clinical studies have shown that ICS use in bronchiectasis is associated with higher rates of pulmonary infection, more frequent exacerbations, and increased mortality [26,27]. Accordingly, current guidelines recommend against routine ICS use in bronchiectasis, except in patients with coexisting asthma [9,28]. ICS not only fail to mitigate the dominant pathogenic mechanisms in bronchiectasis but may exacerbate them, thereby sustaining epithelial injury and promoting carcinogenesis. These mechanistic differences offer a plausible explanation for the opposing associations between ICS use and lung cancer risk in COPD versus bronchiectasis.

*Stenotrophomonas maltophilia* is known to cause chronic infections in approximately 5–15% of patients with non-cystic fibrosis bronchiectasis, similar to *Pseudomonas aeruginosa* [29,30]. Although its prevalence appears consistent across regions, its clinical importance has recently gained attention due to a possible association with lung carcinogenesis. Experimental and clinical studies have suggested that infection *S. maltophilia* may promote tumor progression and poorer prognosis, and that ICS use may increase susceptibility to this pathogen [31,32,33]. Although microbiological analysis was not part of the present study, these findings may provide a rationale for future research exploring infection-related pathways in lung cancer development.

The strengths of this study include the use of propensity score matching to minimize confounding by age, sex, and year of diagnosis, as well as multivariable adjustment for additional factors such as smoking status, BMI, income level, and region of residence. Additionally, by including only patients who initiated ICS therapy after being diagnosed with bronchiectasis, we addressed the temporal relationship between exposure and outcome, thereby reducing the risk of reverse causation.

Several limitations should be acknowledged. First, the claims data lacked detailed clinical information, such as pulmonary function parameters, radiologic findings, and histopathological confirmation, limiting our ability to assess cancer risk by lesion characteristics. Second, ICS exposure was based on prescription codes and may not fully capture adherence or actual drug usage. Third, unmeasured confounders—such as environmental exposures or genetic predisposition—could not be accounted for.

Despite these limitations, this study provides robust, population-based evidence of a positive association between ICS use and lung cancer risk in patients with bronchiectasis. Our findings underscore the need for cautious use of ICS in this population and support the development of personalized treatment strategies that consider underlying disease mechanisms and individual risk profiles.

This study suggests that the use of ICS in patients with bronchiectasis may increase the risk of lung cancer. Future research should aim to clarify this association more precisely and to develop lung cancer prevention strategies utilizing advanced technologies. In particular, by integrating clinical factors such as ICS exposure patterns, frequency of pneumonia, and smoking history with radiomic features extracted from low-dose chest CT scans, as well as molecular biological data including circulating tumor DNA and inflammatory biomarkers, an artificial intelligence based multidimensional risk prediction model could be established to more accurately assess the individual risk of lung cancer. Furthermore, real-time monitoring of medication adherence and symptom changes through smart inhalers or wearable digital health technologies could enable personalized management, such as adjusting ICS dosage, thereby minimizing the risk of lung cancer and improving treatment safety. Ultimately, incorporating AI and digital health technologies into longitudinal studies of bronchiectasis patients may reduce the potential oncogenic risks associated with long-term ICS use and facilitate precision prevention and early detection of lung cancer.

## 5. Conclusions

This nationwide, population-based cohort study demonstrated that the use of ICS in patients with bronchiectasis was linked to a higher likelihood of developing lung cancer, with the association showing a dose–response pattern. Individuals with greater cumulative exposure faced an elevated risk, indicating that both treatment duration and dosage intensity could play critical roles. The increased risk was most prominent among older adults, men, and those with higher body mass index or a smoking history, suggesting that these groups may be particularly susceptible. Overall, the findings highlight the necessity of weighing potential oncologic risks against therapeutic benefits when considering ICS in this population and contribute meaningful evidence that such exposure may be associated with an increased incidence of lung cancer in bronchiectasis.

## Figures and Tables

**Table 1:** Baseline characteristics.

Characteristics	NHIS-NSC		
	Without ICS (n = 3249)	With ICS (n = 1131)	*p*-Value ^†^
Age (years)	78.6 ± 13.4	81.3 ± 10.7	<0.001
35~60	328 (10.1)	42 (3.7)	<0.001
60~70	470 (14.5)	121 (10.7)	
70~	2451 (75.4)	968 (85.6)	
Men	2163 (66.7)	790 (69.9)	0.270
BMI (kg/m^2^)	22.6 ± 3.5	23.6 ± 3.7	0.001
Smoking status			
Never	1644 (50.6)	551 (48.7)	0.046
Former	766 (23.6)	292 (25.8)	
Current	331 (10.2)	91 (8.1)	
Null	508 (15.6)	197 (17.4)	
Smoking (pack-years)	8.3 ± 17.9	10.7 ± 21.2	<0.001
Household income			
<$2000	966 (29.7)	319 (25.2)	0.614
$2000–5000	1139 (35.1)	408 (36.1)	
>$5000	1144 (35.2)	404 (35.7)	
Residential area			
Metropolitan	1518 (46.7)	446 (39.4)	<0.001
Urban	1272 (39.2)	462 (40.9)	
Rural	459 (14.1)	223 (19.7)	
CCI			
0	2980 (91.7)	967 (85.5)	<0.001
≥1	269 (8.3)	164 (14.5)	
COPD *	587 (18.1)	673 (59.5)	<0.001
Asthma *	826 (25.4)	865 (76.5)	<0.001

Data are summarized as number (percentage) for categorical variables and mean ± standard deviation for continuous variables. Abbreviations: BMI, body mass index; CCI, Charlson Comorbidity Index; COPD, chronic obstructive pulmonary disease; ICS, inhaled corticosteroids; NHIS-NSC, national sample cohort of National Health Insurance Service. ^†^ Two-sided chi-squired test and *t*-test where appropriate. * Two or more insurance claims with COPD or asthma disease codes during the study period.

## Data Availability

This study employed data from the National Health Insurance Service (NHIS) subject to licensing restrictions that preclude public dissemination. Access requests must be filed through the NHIS web portal (https://nhiss.nhis.or.kr) and authorized accordingly. Use of the data is restricted to secured remote environments within Korea.

## Abbreviations

The following abbreviations are used in this manuscript:

BMI	Body Mass Index
CCI	Charlson Comorbidity Index
CI	Confidence Interval
COPD	Chronic Obstructive Pulmonary Disease
HR	Hazard Ratio
ICD	International Classification of Diseases
ICS	Inhaled Corticosteroids
IRB	Institutional Review Board
NHIS	National Health Insurance Service
NSC	National Sample Cohort
